# Staging Strategies During Complex Endovascular Aortic Procedures to Minimize Spinal Cord Ischemia Rates: A Narrative Review

**DOI:** 10.3390/jcm14196998

**Published:** 2025-10-03

**Authors:** Alessandro Grandi, Andrea Melloni, Pietro Dioni, Stefano Bonardelli, Luca Bertoglio

**Affiliations:** Division of Vascular Surgery, Department of Clinical and Experimental Sciences, University of Brescia, ASST Spedali Civili of Brescia, 25123 Brescia, Italy; a.grandi004@unibs.it (A.G.); p.dioni001@unibs.it (P.D.); stefano.bonardelli@unibs.it (S.B.)

**Keywords:** complex endovascular aortic repair, thoracoabdominal aortic aneurysm, spinal cord ischemia, staging, FBEVAR, segmental artery embolization, tevar

## Abstract

Endovascular repair of thoracoabdominal aortic aneurysms (TAAAs) requires multidisciplinary expertise to minimize mortality and disabling complications. Despite satisfactory outcomes with this approach being common knowledge, extensive aortic coverage occurring in fenestrated/branched endovascular aortic repair (F/B-EVAR) carries a non-negligible risk of spinal cord ischemia (SCI). Recently, many authors have proposed different endovascular strategies to mitigate the risk of SCI; however, the real effectiveness of these maneuvers is not universally recognized due to a lack of standardized protocols among individual centers. Several adjuncts have been proposed to obtain staged occlusion of segmental aortic branches to promote spinal cord preconditioning. These strategies include proximal thoracic aortic repair (PTAR), temporary aneurysm sac perfusion (TASP), and minimally invasive staged segmental artery coil embolization (MIS^2^ACE). The present paper aims to provide an overview of the most advanced staging strategies used in high-volume aortic centers, pointing out that it takes meticulous preoperative planning to face every clinical scenario.

## 1. Introduction

Repair of extensive thoracoabdominal aortic aneurysms (TAAAs) requires multidisciplinary expertise to minimize the risk of mortality and disabling complications. These procedures pose formidable technical challenges irrespective of the approach. Spinal cord ischemia (SCI) is one of the most serious postoperative complications of TAAA surgery [1,2,3]. Even with the introduction of fenestrated-branched endovascular aortic repair (FB-EVAR), SCI rates still vary between 4.3% and 40% [4,5,6,7]. Although the risk is directly related to the extent of aortic coverage, potential explanations for the wide variation in reported rates include the disparate SCI definitions adopted in various studies, patient heterogeneity, and variations in the use of preventive strategies [8]. Irrespective of the symptoms of presentation (paraparesis up to irreversible paralysis) [9], irreversible neurological damage to the spinal cord can lead to poor quality of life and augmented mortality in this population [10,11].

Multiple strategies have been proposed to reduce the risk of SCI; however, their actual effectiveness is not universally acknowledged due to a lack of homogeneity in the protocols, which very often differ depending on the single center [12,13,14]. Most of the strategies to prevent SCI during endovascular repair focus on improving spinal cord perfusion. Spinal cord perfusion is guaranteed by increasing systemic blood pressure, facilitating oxygen delivery, and decreasing cerebrospinal fluid (CSF) pressure while promoting collateral flow [15,16,17,18,19]. Furthermore, special monitoring techniques and staging procedures have been proposed so far to mitigate SCI incidence [2,4,6]. Previous descriptions addressed the impact of SCI severity on 30-day mortality [20]. In a German study based on insurance data claims of endovascular TAAA repair, the 30-day mortality was significantly higher in the SCI group than the overall patient cohort (23% vs. 8%; *p* < 0.001) and varied by the SCI deficit level: paraplegia, 46%; paraparesis with <50% muscle function, 13%; and paraparesis with >50% muscle function, 0% (*p* = 0.001). The occurrence of SCI was also associated with higher 90-day mortality (15% vs. 1%; *p* < 0.05) and with decreased long-term survival after FB-EVAR for TAAA (hazard ratio, 2.54; *p* < 0.003) [21].

From an anatomical perspective, the spinal cord collateral network includes not only the segmental aortic branches but also collaterals from the vertebral, intercostal, hypogastric, and paraspinal muscular branches [22,23,24]. Results from experimental models have demonstrated that single-stage ligation of the segmental aortic branches is associated with a significant reduction in the spinal cord perfusion pressure, slower recovery to baseline values, and increased rates of paraplegia as compared to a multistage approach [25,26]. In the clinical setting, several strategies have been proposed to achieve controlled occlusion of segmental aortic branches. These strategies include proximal thoracic aortic repair (PTAR), temporary aneurysm sac perfusion (TASP), and minimally invasive staged segmental artery coil embolization (MIS^2^ACE) [27,28,29,30,31,32,33]. A percutaneous approach allows for unlimited procedures [34,35,36]. A limitation of staged approaches is the inherent risk of aortic rupture between staged procedures and the burden of multiple operations [37,38].

The present paper aims to review the principal staging strategies to prevent SCI currently employed in high-volume aortic referral centers. A narrative review of the literature was conducted using PubMed and Google Scholar for articles published between 2015 and 2025, using keywords such as ‘spinal cord ischemia’, ‘spinal cord ischemia prevention,’ and ‘complex endovascular aortic procedures.’ Relevant English-language studies were selected based on their relevance to the review topic.

## 2. Spinal Cord Circulation and Collateral Network

The blood supply to the spinal cord comes from one anterior spinal artery and two posterior spinal arteries that run longitudinally along the cord. These vessels are fed by paired segmental arteries arising directly from the aorta: two or three in the cervical region, two or three in the thoracic region, and none or one in the lumbosacral region [39]. The spinal branches give rise to anterior and posterior radicular arteries that join the anterior and posterior spinal arteries at a few locations. The most dominant anterior radiculomedullary artery in the thoracolumbar area is known as the artery of Adamkiewicz, which is considered the primary blood supply of the spinal cord.

Despite this anatomical division, there is growing experimental and clinical evidence that the presence of a spinal collateral network can compensate for the loss of segmental arteries after aortic surgery [39,40]. This interconnected network of blood vessels is determined by the communication between the anterior spinal artery (i.e., the intraspinous network) and muscular branches providing blood supply to the adjacent muscles of the back (i.e., the paraspinous network). Additionally, multilevel connections between spinal muscles are present and can function as a surrogate blood supply linking adjacent spinal cord segments when the principal input is excluded. These bonds between spinal cord segments can be enhanced by ischemic preconditioning. Intraspinous and paraspinous collateral network rerouting and remodeling is thought to play an important role in maintaining spinal cord blood supply after segmental artery loss.

A deeper understanding of the complex and dynamic collateral network concept derived from animal studies and from clinical anatomical imaging after extensive open surgical repair pioneered by Randal Griepp and Cristian Etz [26,41,42,43,44,45]. Following the loss of segmental arteries, the interspinous network’s diameter increased, and the paraspinous network’s vessels shifted from a metameric orientation that was approximately perpendicular to the spinal cord to one that was parallel to it. This adaptive change occurs very early; in fact, the anterior spinal artery showed an increased diameter after 24 h from the procedure, and after five days, the anterior spinal and epidural arterial network improved in diameter by 80% to 100% (*p* < 0.001) [26]. Although there is no guarantee that these animal models are an accurate reflection of what happens in humans, they show very well the process of spinal arterial network change in mammals after covering aortic segments [26].

Summarizing, the comprehensive elements of the collateral network rely on: (1) the existence of an axial network of small arteries in the spinal canal, paravertebral tissue, and musculature that anastomose among themselves and tributers to the spinal cord [46]; (2) contributions not only from segmental intercostal and lumbar arteries but also from the vertebral (cephalic input) and hypogastric arteries (distal input); and (3) vessel remodeling and reorientation of flow within the collateral network from one source to another upon reduction in selective inflow source [25]. Thus, the collateral network allows for some degree of adaptation to the loss of individual contributors to perfusion until a point beyond which dysfunction is inevitable.

## 3. Spinal Cord Preconditioning and Staging

The first clinical evidence that staging may reduce the risk of mortality and SCI was the observation of more favorable outcomes among patients who had staged open surgical repair of extent II TAAAs [45,47,48,49]. Multistage endovascular repair has gained popularity in the last decade, but evidence remains limited to a few single-center reports. Firstly, The Cleveland group reported a comparison of single- and multistage strategy in 87 patients treated for Extent II and III TAAAs by FB-EVAR, also comparing intentional and unintentional (type I endoleak) staging. In that study, “any SCI” occurred in 38% of single-stage and 11% of multistage procedures [44]. Furthermore, unintentional (OR, 0.02; *p* = 0.014) and intentional staging (OR, 0.01; *p* = 0.019) were both effective in reducing risk of SCI. Among patients who had unintentional staging, there was no added benefit for intentional staging, with a risk of SCI of 1% and 2%, respectively.

Following the first report from the Cleveland Clinic, different groups started to intentionally stage the TAAA repair. Juszczak et al. [50] reported two hundred and seventy consecutive patients with juxtarenal (JRAAA) (*n* = 69) or TAAAs (*n* = 201) who underwent elective FEVAR (*n* = 192) or BEVAR (*n* = 78) with renovisceral stent grafting; of those, non-ambulatory SCI was present in 6 (2.2%). All of them had a supraceliac sealing zone > 40 mm. They reported a significant reduction in SCI after a personalized, selective protocol with liquor drainage was started. Bertoglio et al. [49] analyzed 80 high-risk patients treated by multistage FB-EVAR with mortality of 8% and permanent paraplegia in 5%. Dias-Neto et al. [51] compared outcomes of single- or multistage approaches during complex endovascular aortic repair of extensive TAAAs in 24 centers. A total of 1947 patients underwent FB-EVAR: 155 extent I (10%), 729 extent II (46%), and 713 extent III TAAAs (44%). Among patients undergoing elective repair (*n* = 1597), the composite endpoint of 30-day/in-hospital mortality and/or permanent paraplegia rate occurred in 14% of single-stage and 6% of multistage approach patients (*p* < 0.001). After adjustment with a propensity score, the multistage approach was associated with lower rates of 30-day/in-hospital mortality and/or permanent paraplegia (OR, 0.47; *p* = 0.006) and higher patient survival at one year (87% vs. 79%) and 3 years (73% vs. 64%; adjusted HR, 0.714; *p* = 0.029), compared with a single-stage approach.

A limitation of multistage repair is the risk of interval aneurysm rupture. Even among patients undergoing elective repair, a multistage approach is not suitable for all patients. Patients with large or rapidly expanding aneurysms may be better suited for a single-stage procedure to avoid the risk of rupture [44]. Conversely, some patients might not meet the criteria for final completion, or they might not survive to follow-up. Kasprzak and colleagues reported that five of 40 patients (13%) undergoing TASP were not able to complete the repair due to death, complications, patient refusal, or technical difficulty [29]. In their study, Dias-Neto et al. were not able to obtain an accurate account of patients who failed final completion of the repair [51].

The risk of interval aneurysm rupture was reported in a study of 235 patients treated with patient-specific devices [37,38]. There were 10 patients who suffered interval aneurysm rupture (4%), of whom six had emergent repair with 0% mortality and four who died from aneurysm rupture. The estimated risk of rupture was 6% at six months. Therefore, eligibility for the multistage approach, as well as the ideal staging strategy and timing for completion, needs to be tailored based on the anticipated risk of aneurysm rupture.

The risk of SCI is directly related to the extent of coverage, which is low with Extent IV TAAAs and complex abdominal aneurysms. The pooled rate for SCI for all TAAAs is 4.0%, ranging from 15.0% with extent II and 2.0% with extent IV TAAAs [8]. More extensive aneurysms demonstrate the highest potential benefit for a multistage approach.

Bertoglio et al. [52] analyzed 240 patients from different Italian centers, of whom 43 (18%) had presented with an impaired collateral network, 136 (57%) had had historical staging (previous aortic operations), and 157 (65%) had received procedural staging (dividing a procedure in different steps). Preoperative spinal fluid cerebrospinal drainage was performed in 130 patients (54%). Permanent SCI (grade 3) was observed in 13 patients (5%) and was negatively affected by both an impaired collateral network (OR, 17.3; *p* = 0.016) and the presence of bilateral iliac occlusive disease (OR, 10.1; *p* = 0.046). Both historical (OR, 0.02; *p* = 0.014) and procedural (OR, 0.01; *p* = 0.019) staging mitigated the permanent SCI rates. The development of SCI was associated with the need for postoperative transfusions (OR, 1.4; *p* = 0.014) and the occurrence of postoperative renal complications (OR, 6.5; *p* < 0.001).

During the first decades of thoracic endovascular repair, a previous aortic repair has been considered a risk factor for SCI when the treatment entails coverage of the entire thoracic/thoracoabdominal aorta. Different studies have analyzed the outcomes of complex endovascular aortic repair for patients with prior infrarenal aortic repair and reported permanent SCI rates ranging from 2% to 13% [53,54,55,56]. Moreover, several studies have failed to demonstrate the detrimental role of prior aortic surgery on SC outcomes after F/BEVAR for TAAAs [5,57,58,59,60,61,62,63]. In these studies, when treating extensive TAAAs, the common correlation between SCI and the amount of thoracic coverage included intraoperative bleeding, procedural complexity, and procedural length but not previous aortic surgery. A 2018 study by Kaushik and colleagues [64] showed that prior infrarenal aortic surgery could be protective against SCI after endovascular TAAA repair [9 of 85 (11%) vs. 0 of 68 (0%); *p* = 0.005]. However, the analyzed cohort included 54% with type IV TAAAs or pararenal aneurysms, which, albeit equally distributed between the two study groups, might not allow for the generalization of these results to the repair of type 1–3 TAAAs. In the series analyzed by Bertoglio et al. [52], previous aortic surgery was associated with reduced rates of permanent SCI after F/BEVAR for extent I to III or V TAAAs (OR, 0.02; *p* = 0.014), with a 1.5% incidence of permanent SCI, similar to the previously reported data. An additional finding was that historically staged patients, the protective role of procedural staging was not observed (nonstaged, 1 of 46 [2%]; staged, 1 of 90 [1%]; *p* = 0.626). Upon these results, expedited TAAA repair could be offered to this cohort of patients, avoiding interval rupture without increasing SCI rates.

A particular emphasis should be placed on the role of CSFD along with staging procedures in SCI prevention. Several papers reported a high risk of complications when CSFD is used unselectively in FB-EVAR. Kärkkäinen et al. [65] reviewed 187 patients treated with endovascular aortic repair, reporting 12 patients (6%) with intracranial hypotension, including three (2%) who had intracranial hemorrhage and nine (5%) with post-dural puncture headache requiring blood patches in six. Another six patients (3%) developed spinal hematomas resulting in paraplegia in two (1%) and transient paraparesis in two (1%). Four patients had bleeding during attempted drain placement, which required postponement of F-BEVAR. Technical difficulties were experienced in almost ¼ of drain insertions, more often in patients with degenerative lumbar disease documented by preoperative CT scan than in those without. Fluoroscopic guidance showed a lower rate of technical difficulties compared with the blind approach (9% vs. 28%; *p* = 0.01). CSFD-related complications were deemed to be responsible for 31% of overall spinal cord injuries, thus questioning the protective role of indiscriminate drainage placement.

A recent meta-analysis from Leone et al. [66] including six studies totaling 1079 patients and 730 CSFD placements (all prophylactic except one), revealed a CSFD-related mortality rate of 1.4% and an overall morbidity rate of 25.6%. The overall major, moderate, and minor estimated complication rates were 6.1%, 4.6%, and 26.4%, respectively. Severe complications included intracranial hemorrhage (2.8%), spinal hematoma (1.4%), subarachnoid hemorrhage (1.4%), and CSFD-related neurological deficits (1.1%). A pooled estimate of 11.4% for nonfunctioning drainage was found.

The general trend is shifting from prophylactical to therapeutic drainage policy, giving more importance to procedure planning, including staging techniques, in reducing spinal cord injury rates.

### 3.1. Staging Strategies

#### 3.1.1. Proximal Thoracic Aortic Repair (PTAR)

**Figure 1:**
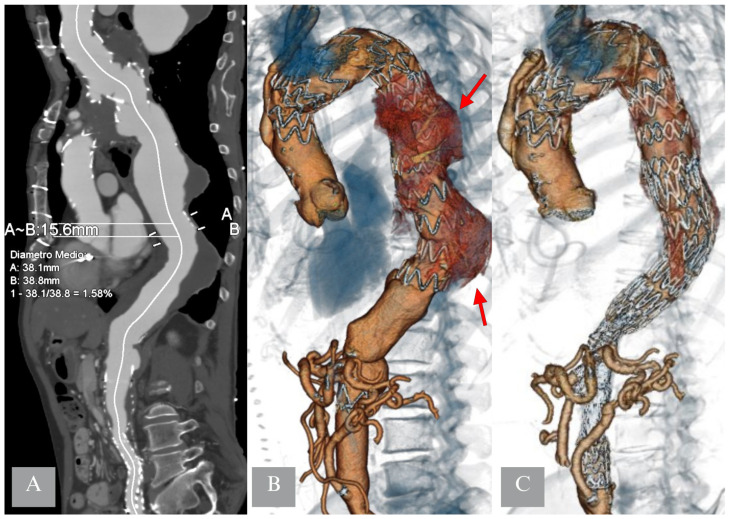
Proximal Thoracic Aortic Repair (PTAR) in a patient with extent I thoracoabdominal aneurysm. An intermediate sealing can be seen at the level of T9 (panel (**A**)), a proximal straight endograft is deployed to promote the thrombosis of the sac in the proximal and mid portion of the descending thoracic aorta (panel (**B**), red arrows), as a second step, completion with a custome-made device for the visceral vessels was performed (panel (**C**)).

**Figure 2:**
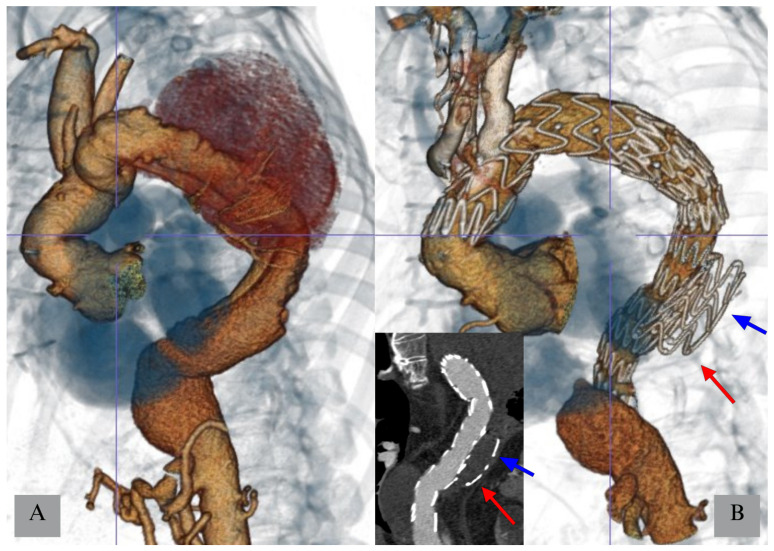
Staged Proximal Thoracic Aortic Repair (PTAR) in a patient with post-dissection extent II thoracoabdominal aneurysm (panel (**A**)) treated with a proximal branched arch graft and a “candy plug” false lume occluder (Cook Medical, blue arrow) inducing complete thrombosis of the dilated false lumen in the proximal thoracic aorta (panel (**B**)). The distal stent of the true lumen and false lumen components should be aligned to guarantee the optimal sealing at this level (red arrows).

Different staging techniques have been described. The most used in cases of extent I-III TAAA is the PTAR, which consists of performing Thoracic Endovascular Aneurysm Repair (TEVAR) as the first operation, with the scope of both creating an adequate proximal landing zone for the visceral component and occluding part of the intercostal arteries and promoting thrombosis of the proximal portion of the aneurysm sac [49], although this technique is not without risks [24]. To do so, anatomical considerations need to be made; in fact, the thrombosis of part of the aneurysm sac can be achieved only if an intermediate distal sealing zone is present (Figure 1). If this was not achievable with a standard TEVAR, in the past it was possible to order custom-made grafts, which were extra tapered to achieve distal sealing in higher diameters, or the “2-in-1 graft”, which allowed for a bigger external diameter while still maintaining a smaller diameter on the inside where the following TEVAR or thoraco-abdominal endograft was placed. In dissection cases, it would help to [67] as it would avoid persistent endoleak in the false lumen. False lumen perfusion would result in a lack of thrombosis of the segmental arteries arising from it, potentially resulting in inconsistent staging (Figure 2). It is crucial not to position the false lumen occluder distally to the thoracic endograft, avoiding false lumen perfusion from entry tears, which might be undetectable at computed tomography angiography. On the other hand, extending the thoracic endograft distally to the false lumen occluder can result in the creation of a stent-graft-induced new entry tear (SINE) [68]. In conclusion, when planning a dissection case in which TEVAR and a false lumen occluder are the staging method, attention should be paid to the selection of accurate distal landing zones for both components, which should possibly be at the same level of the thoracic aorta for the reasons mentioned before.

#### 3.1.2. Temporary Aneurysm Sac Perfusion (TASP)

Temporary aneurysm sac perfusion is performed by deliberately leaving a high-flow endoleak (1B or 1C), typically by noncompletion of one of the branches for the visceral or renal vessels, which remains unbridged until a collateral spinal cord network has developed. The underlying theory is that temporary perfusion of the aneurysm sac will prevent aneurysm sac thrombosis and maintain blood flow through patent intercostal or lumbar arteries, allowing expansion of pre-existing collateral networks or new vessels, reducing the incidence of SCI [29]. The first to introduce this concept was Dr. Ivancev in 2011 by adding paraplegia prevention branches to the design of the endografts [31,32,69]. Of 25 patients who underwent repair of Crawford Extent II or III aneurysms with TASP via aneurysm perfusion branches or noncompletion of branch vessels, five patients with perfusion branches developed temporary SCI with full neurologic recovery, while no permanent deficit was registered. In a series by Kasprzak et al. of 83 patients with TAAA treated with B-EVAR with or without TASP, SCI, or paraplegia, it occurred in 5% of patients with TASP compared with 21% of non-TASP patients who received single-stage repair [29]. There has been no direct comparison between TASP and conventional methods of staged repair. To date, TASP has not been widely adopted, in part due to the potential risk of disseminated intravascular coagulopathy from high flow endoleaks [70], as well as the risk of rupture associated with an induced endoleak. Asymptomatic progression of aneurysm sac diameter (≥5% to 16% growth) has been documented in 26% of patients with TASP intervals >4 weeks, with an estimated risk of rupture during the TASP interval of 3% for all patients and 5% among patients with side branch completion intervals >4 weeks [29].

**Figure 3:**
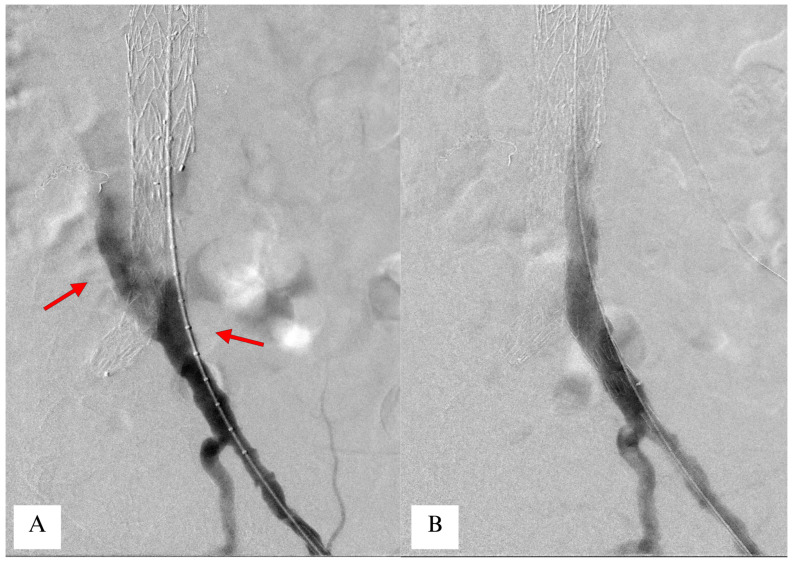
Staging through Temporary Aneurysm Sac Perfusion (TASP). At the end of the thoracoabdominal procedure, the contralateral limb of the distal bifurcated device is left unbridged to maintain a type 1B endoleak (panel (**A**), red arrow). After 10 days, a second staged procedure is performed by completing the aneurysm exclusion with a distal iliac extension component, at completion imaging the endoleak is no longer visible (panel (**B**)).

A variation of the TASP technique has been proposed, in which the unbridged stent-graft component is the contralateral iliac limb [49] (Figure 3). The rationale behind this is that a high-flow type 1B endoleak would reperfuse the sac with fewer adverse events and less risk of sac pressurization due to the flow being directed in the lower limb as well. Furthermore, bridging of the iliac limb is less technically demanding compared to a visceral branch, allowing for possible treatment in non-high-volume aortic centers in case of an emergent rupture.

A third alternative to this approach consists of bridging the one visceral or renal branch inducing a 1C leak with a bare metal stent that would keep the aneurysm sac perfused and ease the recannulation of the target vessel [33].

#### 3.1.3. Selective Coil Embolization of Segmental Arteries (MIS^2^ACE)

**Figure 4:**
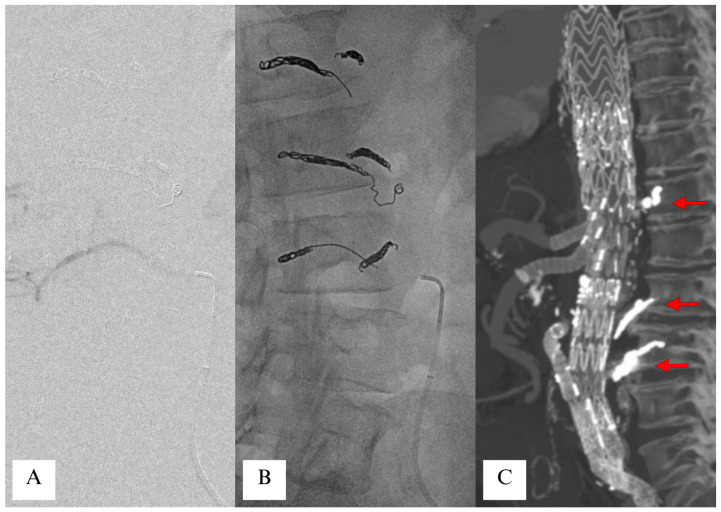
Minimally invasive staged segmental artery coil embolization (MIS^2^ACE). With the aid of fusion imaging, the intended intercostal/lumbar are sequentially catheterized from a percutaneous transfemoral approach (panel (**A**)) and embolized with detachable coils. Up to seven segmental arteries (six in this patient, panel (**B**)) can be embolized in each step of the staged treatment to promote the hyperplasia of the collateral network. Panel (**C**) shows completion of the thoracoabdominal endovascular procedure, featuring the presence of coils in segmental arteries (red arrows).

In selective coil embolization of segmental arteries, as proposed by Dr. Etz and Dr. Kölbel in 2015 [22], intercostal arteries are embolized before endovascular or open thoracoabdominal aortic repair to facilitate “arteriogenic preconditioning” and development of collateral spinal cord perfusion networks [22,26] (Figure 4). In the first published study of this method, a cohort of 57 patients underwent staged coil embolization of a median of five segmental arteries before endovascular repair of Crawford Extent I–IV aneurysms. Of 55 patients who completed definitive endovascular repair, no surviving patients had developed SCI at 30 days [28]. Addas et al. [71] published a retrospective analysis of 17 patients who had an attempted MIS^2^ACE prior to endovascular TAAA repair with a mean follow-up of 350 days. Technically successful embolization occurred in 14 patients (82.4%) and was unsuccessful in three patients. The median number of embolized arteries was 3 and 71% of the target arteries were between T9 and T12. Mean fluoroscopy time was 52 ± 23 min, and mean contrast volume used was 133 ± 56 mL. No complication related to the procedure was registered. The mean interval between embolization and endovascular TAAA repair was 51 days (5–110 days). All patients received spinal drainage at the time of repair. Postoperatively, 2/14 (14%) of patients developed paraparesis in the successful MIS^2^ACE group, and 1/3 (33%) of patients developed paraplegia in the unsuccessful group.

The MIS^2^ACE study, at the time of writing, had just finished enrolling patients [27,72].

Selective coil embolization of segmental arteries can be performed under local anesthesia, enabling continuous monitoring of neurological function and possibly interrupting the procedure in case of spinal cord ischemia symptoms. A thorough evaluation of preoperative computed tomography imaging is critical for identifying open and accessible segmental arteries. Segmental artery occlusion can be achieved with either micro coils, or vascular plugs. A maximum number of seven SAs is currently recommended to be occluded in the same session, and a minimum interval of five days should be awaited between either two MIS^2^ACE sessions or between MIS^2^ACE and the final repair. Adjuvant side effects of MIS^2^ACE include a reduction in segmental back-bleeding during open repair, which can lead to a harmful steal phenomenon, as well as a decrease in the incidence of type II endoleaks during endovascular repair. Current contraindications for MIS^2^ACE are emergency cases [73], hostile anatomy, and a shaggy aorta [74].

Dabravolskaite et al. [75] recently confirmed the safety of the procedure in seven patients, further describing its use also in dissection cases to avoid back-bleeding and type II endoleaks. The authors also performed a meta-analysis on this technique, finding a prevalence of pooled postoperative spinal cord ischemia among MIS^2^ACE patients of 1.9% (95% CI −0.028 to 0.066, *p* = 0.279; 3 studies; 81 patients, 127 coiling sessions). Branzan et al. [76] reported 388 segmental artery occlusions in 54 patients followed by complex endovascular aortic repair, with no in-hospital mortality and one incomplete temporary SCI.

Despite promising preliminary results in terms of safety and efficacy, segmental artery coil embolization can be technically challenging if performed in inexperienced centers due to the need for extensive knowledge of all available ancillary materials and a steep learning curve, which could discourage surgeons in non-referral aortic centers.

A final consideration regarding staging strategies should focus on their pharmacoeconomic impact. Further studies will better clarify which between PTAR, TASP, or MIS^2^ACE performs better as SCI-preventing strategy in each subset of patients-at-risk. Once this is ascertained, a further cost-effectiveness analysis should be performed in order to acknowledge the most sustainable technique. Repeated procedures typically increase the amount of disposable devices employed (introducer sheaths, guidewires, etc.), alongside the costs of repeated hospital admissions in comparison to single-stage procedures. The MIS^2^ACE technique, as compared to TASP and PTAR, comes with additional costs of the occluder devices. The increased costs would be weighed against the tremendous economic burden of each new SCI event, with an average expense of $463,116 per patient in the first postoperative year in a study describing assistance costs in the United States [77].

### 3.2. Theoretical Comparison of the Different Strategies

In Table 1 it has been summarized a theoretical comparison of the different strategies with possible advantages and disadvantages.

## 4. Conclusions

Staging of complex endovascular aortic repair should be considered whenever possible, since it has demonstrated safety and feasibility, decreased rates of spinal cord injury, and a low incidence of interval adverse events. It improved the results of complex endovascular aortic repair, and the staged approach emerged as a safer alternative compared to standard open repair [78]. Different staging processes exist, each having distinct advantages and limitations, some of which remain under investigation; thus, additional studies with direct comparisons are needed to ascertain the most effective strategy.

## Figures and Tables

**Table 1:** Summary of a theoretical comparison of the different strategies with possible advantages and disadvantages. PTAR, proximal thoracic aortic repair; TASP, temporary aneurysm sac perfusion.

Strategy	Preferred Pathology	Advantages	Disadvantages
PTAR	Extent I–III TAAA, dissections	Rapid proximal thrombosis, technically easy	Extensive coverage, possible thrombus embolization
TASP (branch)	Extent I–IV TAAA, dissections	Can be used a dedicated branch	Technically demanding, especially in urgent cases
TASP (iliac)	Extent I–IV TAAA, dissections	Technically easy, especially in emergent cases	-
MIS^2^ACE	Extent I–III TAAA, dissections	May reduce endoleak rates	Technically demanding, may require different intervention

## Data Availability

No new data was created with this paper.
